# Supplementary material for: Cu-Doped Porous Carbon Derived from Heavy Metal-Contaminated Sewage Sludge for High-Performance Supercapacitor Electrode Materials
Source: Nanomaterials (Basel). 2019 Jun 17;9(6):892. doi: 10.3390/nano9060892 (PMC6630645; doi:10.3390/nano9060892)
Supplement: Supplementary file 1 [file nanomaterials-09-00892-s001.pdf]

nanomaterials

Supplementary Information

Article

Fabrication of Cu-based Nanocomposites by Direct Pyrolysis of Heavy Metal-Contaminated Sewage Sludge for Energy Storage System

Zhouliang Tan [1], Feng Yu [1], Liu Liu [1], Xin Jia [1], Yin Lv [1], Long Chen [1], Yisheng Xu [1,2], Yulin Shi [1,*], Xuhong Guo [1,2]

1 Key Laboratory for Green Processing of Chemical Engineering of Xinjiang Bingtuan, School of Chemistry and Chemical Engineering, Shihezi University, Shihezi 832003, P.R. China. 2260049182@qq.com (Z.T.); yufeng05@mail.ipc.ac.cn (F.Y.); 1732619521@qq.com (L.L.); jiaxin_shzu@foxmail.com (X.J.); ag_125@163.com (Y.L.); chenlong2012@sinano.ac.cn (L.C.); ecustn@gmail.com (Y.X.); shiyulin521@126.com (Y.S.) and guoxuhong@ecust.edu.cn (X.G.)

2 State Key Laboratory of Chemical Engineering, East China University of Science and Technology, Shanghai 200237, P.R. China

* Correspondence: shiyulin521@126.com (Y.S.); Tel.: +86-993-2055030

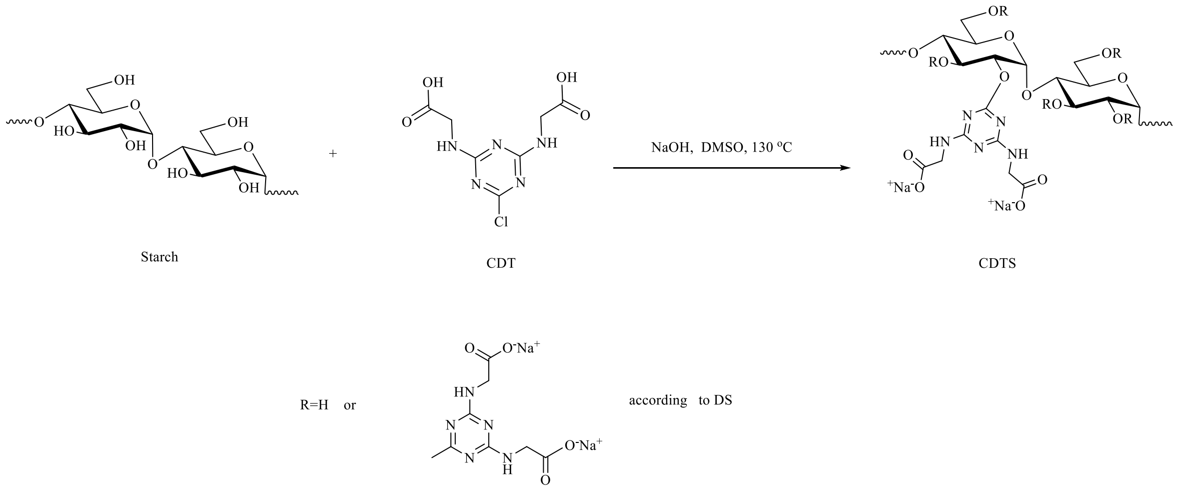

Figure S1. Starch-based flocculant containing ionizable carboxyl group was synthesized using 2-chloro-4,6-diglycino-[1,3,5]-triazine (CDT) as the etherification agent.

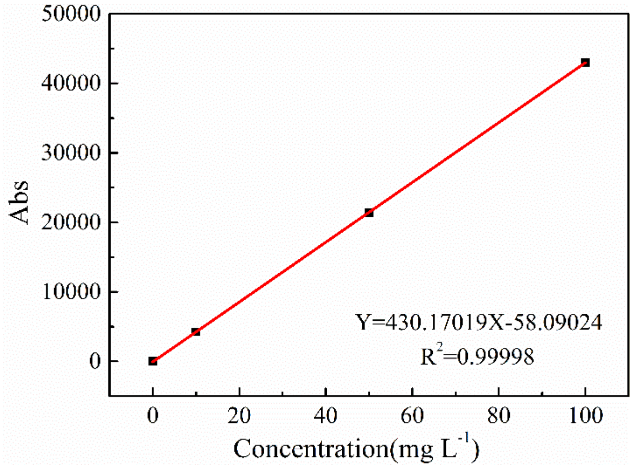

Figure S2. Standard curve of Copper.

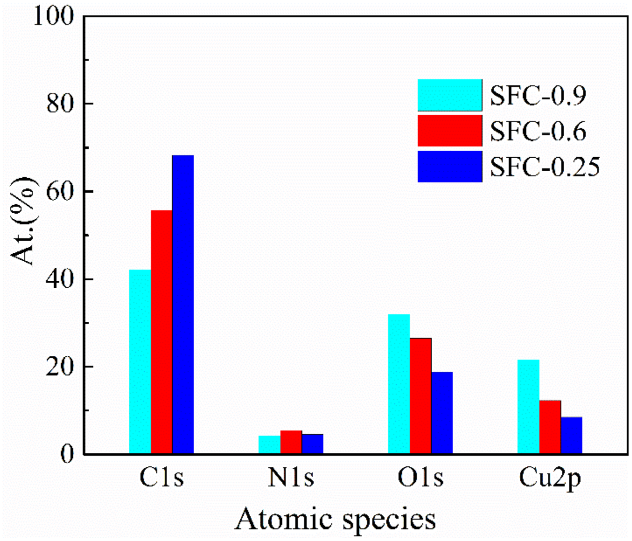

Figure S3. The elemental analysis of SFC-x.

Table S1. Different types of carbon bonds.

Sample	C=O	C-N/C-O	C=C-C
SFC-0.9	30.7%	34.15%	35.15%
SFC-0.6	29.93%	33.38%	36.69%
SFC-0.25	28.8%	34.13%	37.07%

Table S2. Physical parameters for SFC-x.

Sample	R (%)	Q ($mg\ mg^{-1}$)	S_{BET} ($m^2\ g^{-1}$)	S_{mic} ($m^2\ g^{-1}$)	S_{mes} ($m^2\ g^{-1}$)	Pore Volume (%)	
						V<2nm	V>2nm
SFC-0.9	45.02	0.9	68.54	48.31	20.23	54.43%	45.57%
SFC-0.6	99.50	0.6	258.48	232.56	25.92	77.65%	22.35%
SFC-0.25	50.49	0.25	285.24	275.68	9.56	91.61%	8.39%

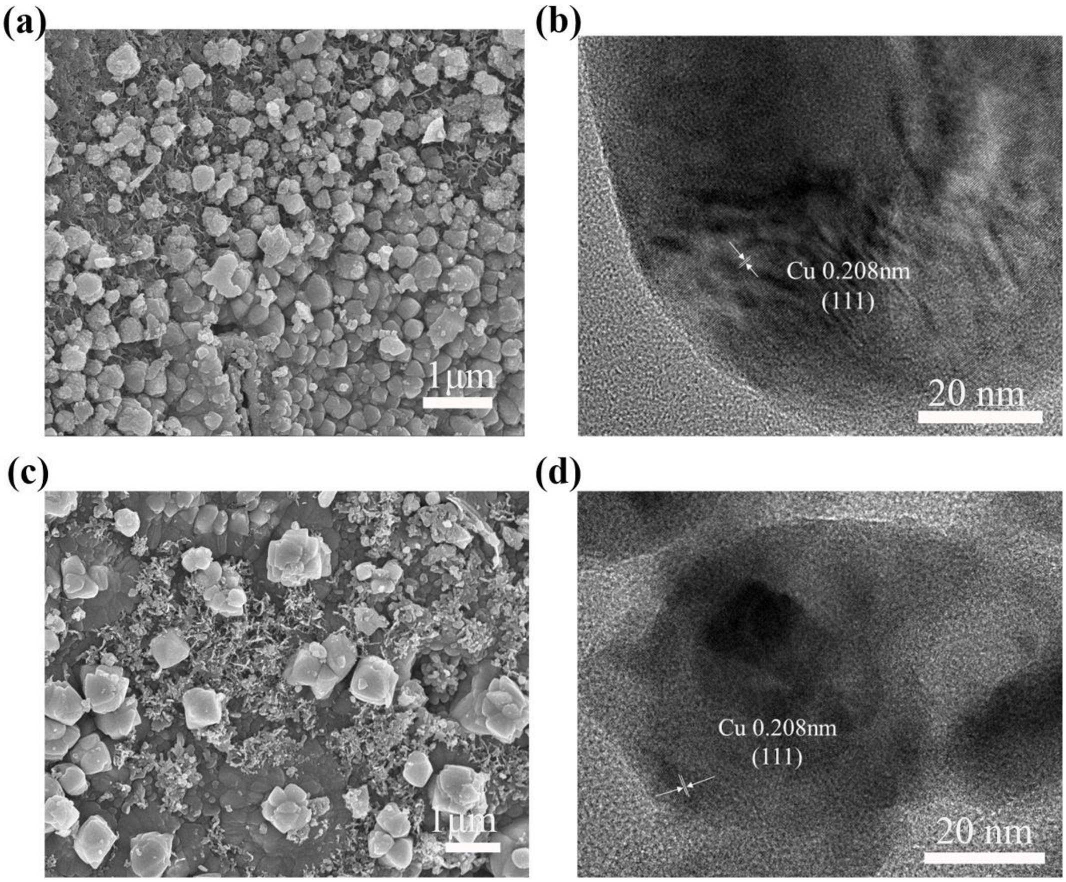

Figure S4. (**a**) SEM images and (**b**) HRTEM of SFC-0.9. (**c**) SEM images and (**d**) HRTEM of SFC-0.25.

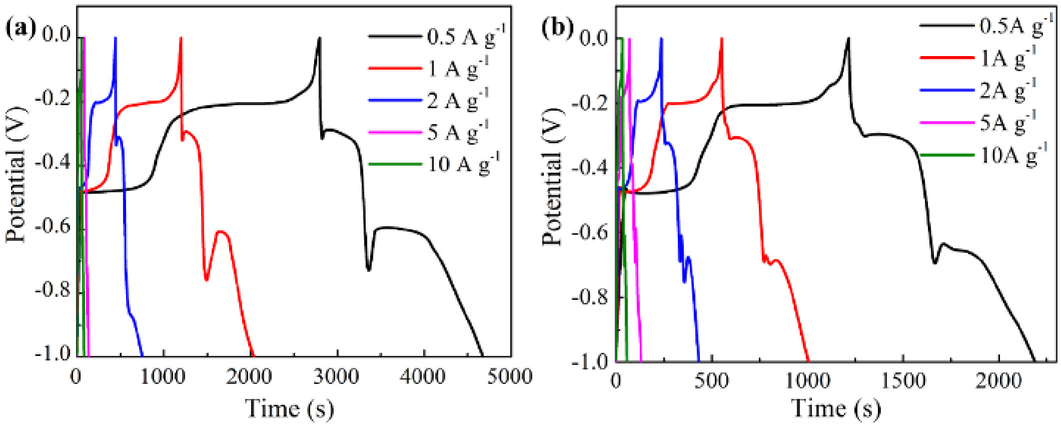

Figure S5. GCD curves of (**a**) SFC-0.9 and (**b**) SFC-0.25 electrode at different current densities in three-electrode.

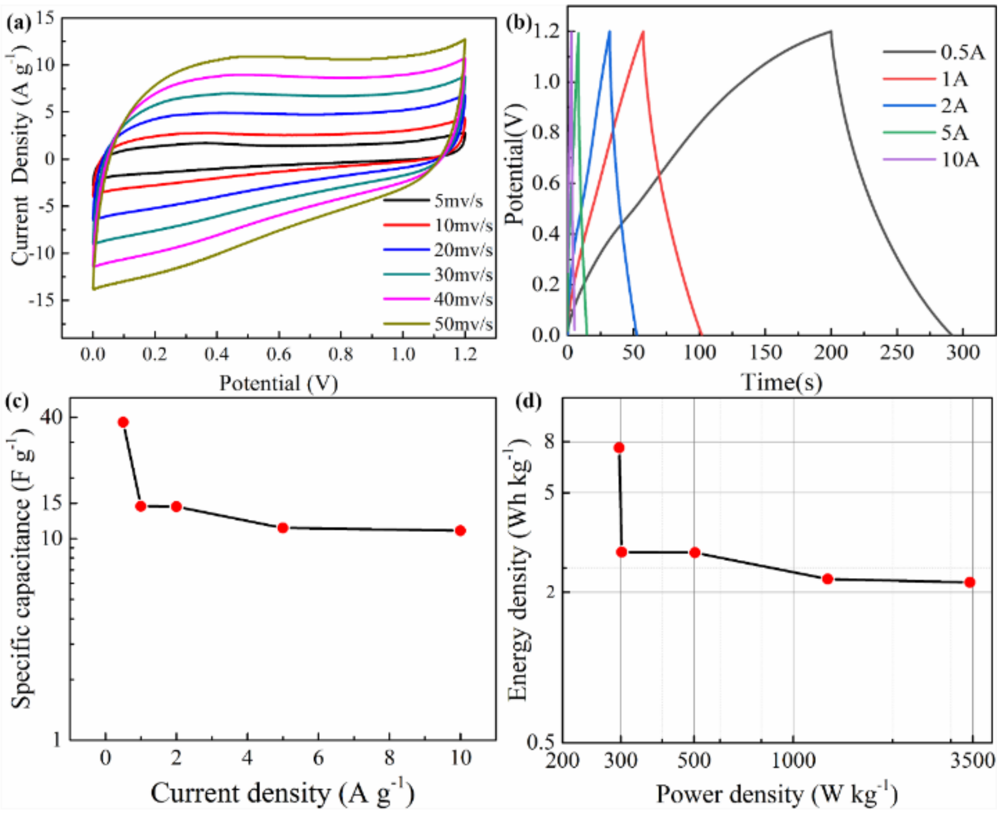

Figure S6. (**a**) CV of SFC-0.6 at various scan rates (5–50 mV s^{-1}) in two-electrode (**b**) GCD curves of SFC-0.6 electrode at various current densities (0.5–10 A g^{-1}) in two-electrode. (**c**) The specific capacitance of various electrodes as a function of current density based on the GCD curves. (**d**) Energy density with respect to the power density of the SFC-0.6.

The energy densities and power densities were calculated from the following (Equations 1 and 2) [1]:

$$E = \frac{1}{2} C_m (\Delta V)^2 \quad (1)$$

$$P = \frac{\mathrm{E}}{\Delta \mathrm{t}} \quad (2)$$

where E (Wh kg^{-1}) is the energy density, P (W k g^{-1}) is the power density, C_m (F g^{-1}) is the capacitance of the capacitor, ΔV (V) is the operating potential window, and Δt (s) is the discharge time.

References

1. Teng, S.; Siegel, G.; Prestgard, M.C.; Wang, W.; Tiwari, A. Synthesis and characterization of copper-infiltrated carbonized wood monoliths for supercapacitor electrodes. *Electrochim. Acta* **2015**, 161, 343-350.
